# Association between plasma concentrations of branched-chain amino acids and adipokines in Japanese adults without diabetes

**DOI:** 10.1038/s41598-018-19388-w

**Published:** 2018-01-18

**Authors:** Ryoko Katagiri, Atsushi Goto, Sanjeev Budhathoki, Taiki Yamaji, Hiroshi Yamamoto, Yumiko Kato, Motoki Iwasaki, Shoichiro Tsugane

**Affiliations:** 1 Epidemiology and Prevention Group, Centre for Public Health Sciences, National Cancer Centre, Tokyo, Japan; 2 Research Institute for Bioscience Products & Fine Chemicals, Ajinomoto Co., Inc, Kawasaki, Japan; 3 Institute for Innovation, Ajinomoto Co., Inc., Kawasaki, Japan

## Abstract

Previous studies have consistently reported an association between circulating levels of branched-chain amino acids (BCAAs) or adipokines and insulin resistance; however, the association between BCAA and adipokine levels remains to be clarified. In this cross-sectional study involving 678 participants (435 men) without diabetes, plasma BCAA (valine, leucine, and isoleucine), adipokine (total and high molecular weight [HMW] adiponectin, leptin, and tumor necrosis factor-α [TNF-α]) concentrations, and an updated homeostasis model assessment of insulin resistance (HOMA2-IR) were measured. The association between the concentrations of total BCAAs and adipokines was adjusted for confounding factors, including body mass index. For the lowest and highest BCAA quartiles, the adjusted geometric mean levels of HMW adiponectin were, respectively, 1.51 and 0.91 μg/mL, in men (P for trend < 0.0001); 3.61 and 2.29 μg/mL, in women (P = 0.0005). The corresponding geometric mean levels for leptin were 1681 and 2620 pg/mL, in men (P = 0.003), and 4270 and 6510 pg/mL, in women (P = 0.003). Those for HOMA2-IR were 0.89 and 1.11, in men (P < 0.0001), and 0.79 and 0.96, in women (P < 0.0001); no significant association was found with TNF-α. These results suggest significant associations between BCAA concentrations and those for adiponectin, leptin and HOMA2-IR in individuals without diabetes.

## Introduction

Obesity and metabolic diseases have reached epidemic proportions, globally^1^. To prevent these, the underlying mechanisms of obesity and its related diseases have been investigated. Recently, adipose tissue has started to be considered an endocrine organ that secretes metabolism-related bioactive mediators, called adipokines^2,3^, rather than a simple lipid storage depot. Adipokines are involved in systemic inflammation and insulin resistance^3,4^. Further, some adipokines, including tumour necrosis factor-α (TNF-α), act as pro-inflammatory mediators^5^, whereas another, adiponectin, is recognized as an anti-inflammatory cytokine and its disruption causes insulin resistance^6,7^. Adiponectin includes three forms, low molecular weight, middle molecular weight, and high molecular weight (HMW). Of these, HMW adiponectin is thought to play major roles in improving insulin resistance^8^. Leptin, another type of adipokine, has anti-diabetic effects, but because leptin resistance develops in obese individuals, high circulating levels of leptin are often observed in such individuals^4,9^.

Recent studies have utilized metabolic profiling to investigate metabolites that may be relevant in metabolic diseases^10^. Branched-chain amino acids (BCAAs) are essential amino acids that include valine, leucine, and isoleucine. Recent studies have reported that circulating BCAA-related metabolites are positively associated with insulin resistance^11,12^. According to a systematic review, the positive association between circulating BCAA levels and insulin resistance, as assessed by homeostasis model assessment of insulin resistance, is consistently reported in studies from various countries and involving participants of varying ethnicities^12^. Furthermore, in prospective cohort studies, including the Framingham Offspring Study, high plasma BCAA concentrations were linked to an increased risk of type 2 diabetes^11,13^. Several hypotheses have sought to explain the association between BCAAs and insulin resistance or diabetes risk; however, the precise mechanisms remain largely unknown.

Although both adipokine and BCAA levels are associated with insulin resistance^3–8,12^, there is little evidence of an association between BCAAs and adipokines in humans. Clarifying the association between BCAAs and adipokines may provide further insight into the linkage among these three factors. One study involving patients with type 2 diabetes reported a relationship among BCAAs, insulin-related markers, and adiponectin levels^14^; however, investigating the association in a non-diabetic population is crucial because BCAA, adipokines, and insulin resistance are already dysregulated in patients with diabetes. Therefore, in the present study, we investigated the association of plasma BCAA levels with adipokines and C-peptide levels (markers of insulin resistance) in middle-aged Japanese individuals without diabetes.

## Methods

### Study population

Participants in this study comprised the control group from the Colorectal Adenoma Study in Tokyo (CAST); study details are described elsewhere^15–17^. Briefly, individuals undergoing colonoscopic cancer screening at the Research Centre for Cancer Prevention and Screening, National Cancer Centre, Tokyo, Japan, between February 2004 and February 2005, were recruited into the study. Eligible participants included men (50–79-years-old) and women (40–79-years-old) without known histories of colorectal diseases, including colorectal adenoma, ulcerative colitis, Crohn’s disease, familial adenomatous polyposis, carcinoid tumours, or any malignant neoplasm, and who had not undergone colectomies^15,16^. Among the 3,212 individuals undergoing magnified colonoscopy, 2,234 were eligible; 526 men and 256 women had at least one adenomatous polyp and were included in the adenoma case group. Among the remaining 1,452 participants, 482 men and 721 women were deemed to be potential controls due to the absence of other benign lesions, such as hyperplastic polyps, inflammatory polyps, or diverticula. Among the men, the number of cases was larger than the number of potential controls; therefore, all potential controls were included in the control group. For efficiency, frequency-matching was conducted among the women, using 10-year age range categories and two screening periods (first and second halves). Finally, 482 men and 256 women who were included as the control cohort in the CAST study were also included in the present study. All of these individuals provided written informed consent for participation in the study; the study protocol was approved by the institutional review board of the National Cancer Centre, Tokyo, Japan (No. G16-03), in accordance with relevant ethical guidelines in the field of medical research in Japan and the study was performed in accordance to the guidelines according to the Declaration of Helsinki.

### Questionnaire and anthropometric measurements

All participants were asked to complete a self-administered questionnaire before the screening examination. This questionnaire surveyed lifestyle information, such as past and current medical and treatment histories, cigarette smoking habits, alcohol consumption, physical activity, and also included a food frequency questionnaire related to 145 food and beverage items. Details of the food frequency questionnaire are described elsewhere^18^. Body heights and weights, measured by medical personnel, were used to calculate body mass indexes (BMIs), defined as the body weight (kilograms) divided by the square of the body height (metres). Visceral fat volume was calculated using a software (Fujifilm Medical, Tokyo, Japan). Visceral fat area was first calculated from multi-slice computed tomography images and the visceral fat volume quantified from these integrated images^16^.

### Laboratory assays

Blood sample collections were scheduled one day before the screenings. Fasting (>8 h, confirmed in 75% of the individuals; 4% had fasted for <6 h, with a minimum fast of 4 h) venous blood was collected into vacutainer tubes, containing ethylenediaminetetraacetic acid, centrifuged, frozen at −80 °C, and preserved until analysed.

We measured C-peptide levels only and did not assess insulin levels in this study. C-peptide is a robust measure of insulin secretion^19^; thus, we considered it to be a suitable marker in this epidemiological study. Moreover, the half-life of C-peptide is longer than that of insulin and the rate of C-peptide clearance is constant and slower than that of insulin^20,21^.

Plasma concentrations of BCAAs (valine, leucine, and isoleucine), adipokines (total and HMW adiponectin, leptin, and TNF-α), and C-peptides were measured in the preserved samples. Detailed measurement methods and reported intra-assay coefficients of variation for adiponectin, leptin, TNF-α, C-peptide and BCAA levels were described previously^22–24^. Briefly, total and HMW adiponectin levels were measured using an enzyme-linked immunosorbent assay (ELISA) with Human Adiponectin ELISA Kit for Total and Multimers (Sekisui Medical, Tokyo, Japan) at Mitsubishi Chemical Medience (Tokyo, Japan). For both total and HMW adiponectin assays, the limit of detection was 0.39 μg/mL. Plasma concentrations of leptin and TNF-α were measured using the Human Serum Adipokine (Panel B) LINCOplex Kit (Millipore, Billerica, MA, USA) at GeneticLab (Hokkaido, Japan). The minimum detection level for leptin was 85.4 pg/mL and that for TNF-α was 0.14 pg/mL. Plasma C-peptide concentrations were measured using a Fujirebio (Tokyo, Japan) reagent and a chemiluminescent enzyme immunoassay method at SRL (Tokyo, Japan); the reported minimum detection level was 0.04 ng/mL. BCAA levels were measured using the UF-Amino Station and a liquid chromatography/mass spectrometry (Shimadzu, Kyoto, Japan) method at the Ajinomoto Institute for Innovation (Kanagawa, Japan). Plasma glucose levels were measured at the time of the cancer screening in the National Cancer Center hospital laboratory using Quick-auto (Shino-test, Tokyo, Japan), based on the hexokinase method in the automatic analyzer (Hitachi 7600, Hitachi, Tokyo, Japan).

### Statistical analysis

Among the 738 participants identified as controls in the CAST study, those who reported a history of diabetes (n = 47), were currently taking medications for diabetes (n = 6), had blood glucose levels > 200 mg/dL (n = 2), had missing BCAA values (n = 2), or demonstrated BCAA outliers (defined as BCAA concentrations > 3 standard deviations above or below the age and sex standard) (n = 3) were excluded. Ultimately, 678 participants (435 men and 243 women) were included in the analysis.

Some participants had levels of HMW adiponectin (45 men, 2 women) or leptin (27 men, 1 woman) that were below the level of detection. Although many statistical methods have been developed to deal with nondetect data^25^, a common method was used in this study. Therefore, such nondetect data were replaced with half the threshold values (HMW adiponectin = 0.195 $(0.39\times \frac{1}{2})$ µg/mL, leptin = 42.7 $(85.4\times \frac{1}{2})$ pg/mL). Sensitivity analyses, excluding individuals below the detection limit, were also conducted.

The BCAA concentrations were calculated as the sums of the valine, leucine, and isoleucine concentrations, and participants were divided into quartiles according to sex-related BCAA levels. Differences in participant characteristics in the quartiles were tested using analysis of variance for continuous variables and Chi-square test and Fisher’s exact test for categorical variables. An updated homeostasis model assessment of insulin resistance (HOMA2-IR) was calculated from C-peptide and glucose levels, using the HOMA Calculator version 2.2.3 (https://www.dtu.ox.ac.uk/homacalculator/). Calculation of HOMA2-IR was conducted in participants with 8 hours or more of fasting (n = 298 in men and 208 in women). Adipokine, HOMA2-IR and C-peptide levels were log-transformed to enhance compliance with normality assumptions. Pearson’s correlation coefficients were calculated to examine the associations between BCAA levels and glucose levels, log-transformed adipokine levels, log-transformed C-peptide levels, and log-transformed HOMA2-IR. Two substantial outliers were observed among the men for TNF-α (crude values of 55.2 and 179.2 pg/mL; others were <12 pg/mL), and these were excluded to calculate the correlation between BCAA and TNF-α levels.

For multivariate analysis, adipokines, HOMA2-IR and C-peptide levels were again log-transformed and used as dependent variables in the individual, generalized linear models. The geometric means and 95% confidence intervals were calculated in these models. Potential confounding factors, including age (continuous), physical activity level (quartile), and fasting time (categorized as <8 h or >8 h) were included in Model 1; BMIs (continuous) were included in Model 2. Since HOMA2-IR was calculated in fasting participants, fasting time was not included in Model 1 and 2 for HOMA2-IR. All analyses were performed using SAS, version 9.3 (SAS Institute, Cary, NC, USA). Two-sided p-values < 0.05 were considered statistically significant.

### Data availability

To comply with our privacy and data security policies, the data of the current study are available only for researchers who meet our criteria for access to confidential data. For researchers who have an interest in using the data, please contact Dr. Shoichiro Tsugane at the Center for Public Health Sciences, National Cancer Center, Japan (e-mail address: stsugane@ncc.go.jp).

## Results

Table 1 shows the study population’s basic characteristics, stratified by plasma BCAA quartile and sex. The BMI was significantly different among quartiles among both men and women. No significant differences were observed in other variables, including physical activity, smoking status, or in energy and protein intakes calculated from the food frequency questionnaire. The total and individual BCAA concentrations were inversely correlated with the total and HMW adiponectin levels, and positively correlated with leptin, HOMA2-IR and C-peptide levels (Table 2). Glucose and TNF-α concentrations were not correlated with plasma BCAA levels.

**Table 1:** Basic characteristics of 435 men and 243 women participating in the study, according to their branched-chain amino acid (BCAA) level quartile.

	All	BCAA^4^ quartile (Q)				*P*_*value*_^5^
		Q1	Q2	Q3	Q4	
Men (n)	435	109	109	109	108	
Plasma amino acid level, Median (IQR)						
Plasma isoleucine (μmol/L)	65.6 (58.2–73.4)	54.2 (49.8–58.1)	62.0 (58.8–64.6)	69.7 (65.9–73.3)	78.8 (73.2–86.1)	—
Plasma leucine (μmol/L)	129.5 (118.0–142.7)	110.0 (101.9–117.6)	124.6 (118.3–129.1)	135.7 (130.3–140.9)	151.2 (144.0–161.2)	—
Plasma valine (μmol/L)	237.1 (216.6–262.9)	200.6 (186.3–213.0)	226.7 (220.4–232.7)	247.3 (239.8–254.8)	281.0 (270.5–296.2)	—
Plasma BCAA (μmol/L)	430.9 (396.0–476.0)	368.2 (341.0–385.6)	414.7 (406.3–421.4)	454.7 (443.2–464.0)	510.0 (490.3–540.2)	—
Age (y)	60.0 (5.7)	60.0 (5.9)	59.4 (5.2)	60.2 (6.2)	60.2 (5.6)	0.72
Body mass index (kg/m^2^)	23.5 (2.7)	22.0 (2.6)	23.5 (2.5)	23.9 (2.7)	24.6 (2.2)	<0.0001
≥25^1^ (%)	26.4	11.0	27.5	25.7	41.7	<0.0001
Visceral fat volume (cm^3^)^2^	3296 (1414)	2621 (1291)	3241 (1351)	3409 (1503)	3882 (1227)	<0.0001
Physical activity^3^ (METs-h/d)	35.7 (6.5)	36.4 (6.8)	35.8 (6.1)	34.8 (6.6)	35.9 (6.5)	0.34
Smoking status						0.06
Current (%)	13.1	9.2	12.8	21.1	9.3	
Past (%)	53.1	59.6	54.1	49.5	49.1	
Never (%)	33.8	31.2	33.0	29.4	41.7	
Alcohol consumption						0.66
>300 g/wk, (%)	23.2	25.7	24.8	21.1	21.3	
1–299 g/wk (%)	61.8	61.5	61.5	62.4	62.0	
Non-drinker (%)	14.9	12.8	13.8	16.5	16.7	
Dietary intake						
Total energy (kcal/d)	1995 (606)	1990 (536)	1956 (525)	2044 (665)	1989 (688)	0.76
Total protein (g/d)	67.9 (24.6)	66.6 (20.4)	66.5 (22.2)	69.2 (26.2)	69.2 (29.0)	0.73
Fasting time (h)	11.5 (4.0)	12.1 (4.1)	11.0 (4.2)	11.2 (4.1)	11.6 (3.7)	0.22
Plasma glucose (mg/dl)	97.8 (9.5)	97.9 (8.9)	96.7 (8.5)	97.5 (9.3)	99.1 (10.9)	0.30
C-peptide (ng/mL)	1.36 (0.6)	1.14 (0.5)	1.29 (0.5)	1.38 (0.5)	1.63 (0.6)	<0.0001
HOMA2-IR^4^	1.04 (0.4)	0.88 (0.4)	0.94 (0.4)	1.06 (0.4)	1.27 (0.4)	<0.0001
Women (n)	243	61	61	61	60	
Plasma amino acid level, Median (IQR)						
Plasma isoleucine (μmol/L)	50.1 (44.6–56.9)	42.0 (39.0–45.2)	47.0 (44.1–50.0)	53.0 (49.4–59.4)	60.3 (57.6–63.2)	—
Plasma leucine (μmol/L)	102.8 (91.6–114.2)	86.5 (80.8–91.0)	97.6 (93.4–102.1)	108.9 (104.0–113.3)	123.0 (115.2–129.3)	—
Plasma valine (μmol/L)	193.3 (176.1–220.0)	165.3 (152.5–170.5)	187.6 (181.4–192.1)	201.8 (196.3–211.3)	237.1 (228.2–253.7)	—
Plasma BCAA (μmol/L)	345.8 (316.7–388.4)	296.3 (279.9–306.6)	331.6 (324.7–337.4)	365.2 (351.9–376.3)	413.7 (402.5–439.3)	—
Age (y)	59.5 (6.4)	59.6 (6.4)	58.6 (7.7)	59.0 (5.4)	60.9 (5.9)	0.22
Body mass index (kg/m^2^)	22.2 (2.5)	21.0 (2.2)	21.7 (2.1)	22.8 (2.5)	23.2 (2.8)	<0.0001
Obesity^1^ (%)	12.4	4.9	4.9	23.0	16.7	0.003
Visceral fat volume (cm^3^)^2^	1984 (847)	1620 (659)	1840 (776)	2158 (867)	2382 (907)	<0.0001
Physical Activity^2^ (METs-h/d)	38.0 (9.5)	37.5 (8.9)	39.0 (11.5)	37.3 (7.8)	38.0 (9.3)	0.75
Smoking status						0.99
Current (%)	2.9	3.3	3.3	1.6	3.3	
Past (%)	9.1	8.2	11.5	8.2	8.3	
Never (%)	88.1	88.5	85.3	90.2	88.3	
Alcohol consumption						0.70
>300 g/wk (%)	3.3	6.6	3.3	1.6	1.7	
1–299 g/wk (%)	44.4	39.3	49.2	47.5	41.7	
Non-drinker (%)	52.3	54.1	47.5	50.8	56.7	
Dietary intake						
Total energy (kcal/d)	1869 (628)	1860 (603)	1798 (706)	1822 (571)	1998 (619)	0.30
Total protein (g/d)	72.5 (28.5)	74.4 (28.3)	69.3 (31.4)	69.6 (27.6)	76.9 (26.2)	0.38
Fasting time (h)	13.1 (3.1)	13.6 (2.8)	13.0 (2.9)	13.0 (3.0)	12.7 (3.7)	0.43
Plasma glucose (mg/dl)	96.2 (10.3)	95.4 (8.4)	94.7 (9.4)	96.1 (8.5)	98.7 (13.8)	0.16
C-peptide (ng/mL)	1.15 (0.4)	1.06 (0.3)	1.09 (0.3)	1.13 (0.3)	1.32 (0.5)	0.0003
HOMA2-IR^4^	0.88 (0.3)	0.80 (0.3)	0.82 (0.2)	0.86 (0.3)	1.05 (0.4)	<0.0001

Values are means (SD) except variables for numbers (n) and percentages (%) and plasma amino acid levels.

^1^Obesity is defined as body mass index (BMI) ≥ 25. The number of participants with BMI ≥ 30 were 4 (0.9%) men and 2 (0.8%) women.

^2^Visceral fat volumes were qualified in 338 men and 189 women.

^3^There were 3 missing physical activity values for the men and 3 for the women.

^4^Updated homeostatic model assessment of insulin resistance (HOMA2-IR) was calculated from C-peptide and glucose levels using the HOMA Calculator version 2.2.3 (https://www.dtu.ox.ac.uk/homacalculator/) in participants with fasting time over 8 hours. (n = 298 in men and 208 in women).

^5^BCAA was calculated as the sum of isoleucine, leucine, and valine.

^6^Statistical differences were assessed among quartiles using analysis of variance for continuous variables and Chi-square tests for categorical variables. For categorical variables in women, Fisher’s exact test was used because the number of participants in a few categories was <5.

**Table 2:** Pearson’s correlation coefficients between plasma branched-chain amino acid (BCAA) concentration and insulin resistance-related biomarkers^1^.

	BCAA		Isoleucine		Leucine		Valine	*P* value
	r	*P* value	r	*P* value	r	*P* value	r	
Men								
Glucose	0.08	0.08	0.03	0.47	0.08	0.10	0.09	0.05
Total adiponectin	−0.31	<0.0001	−0.31	<0.0001	−0.30	<0.0001	−0.27	<0.0001
HMW adiponectin	−0.30	<0.0001	−0.30	<0.0001	−0.30	<0.0001	−0.26	<0.0001
Leptin	0.29	<0.0001	0.28	<0.0001	0.25	<0.0001	0.29	<0.0001
TNF-α^2^	0.04	0.47	0.08	0.10	0.04	0.40	0.01	0.78
C-peptide	0.35	<0.0001	0.35	<0.0001	0.29	<0.0001	0.35	<0.0001
HOMA2-IR^3^	0.38	<0.0001	0.37	<0.0001	0.33	<0.0001	0.37	<0.0001
Women								
Glucose	0.16	0.01	0.14	0.03	0.14	0.03	0.17	0.008
Total adiponectin	−0.27	<0.0001	−0.22	0.0007	−0.27	<0.0001	−0.26	<0.0001
HMW adiponectin	−0.27	<0.0001	−0.22	0.0006	−0.28	<0.0001	−0.26	<0.0001
Leptin	0.33	<0.0001	0.26	<0.0001	0.32	<0.0001	0.32	<0.0001
TNF-α	0.11	0.08	0.09	0.17	0.12	0.07	0.10	0.11
C-peptide	0.25	<0.0001	0.15	0.02	0.17	0.006	0.29	<0.0001
HOMA2-IR^3^	0.27	<0.0001	0.22	0.002	0.20	0.004	0.29	<0.0001

^1^Total adiponectin, HMW adiponectin, leptin, TNF-α, C-peptide and HOMA2-IR values were log-transformed.

^2^Among the men, 2 substantial TNF-α outliers (crude values, 55.2 and 179.2 pg/mL) were observed and excluded; others were less than 12 pg/mL.

^3^Updated homeostatic model assessment-insulin resistance (HOMA2-IR) was calculated from C-peptide and glucose levels using the HOMA Calculator version 2.2.3 (https://www.dtu.ox.ac.uk/homacalculator/) in participants with fasting time over 8 hours. (n = 298 in men and 208 in women).

TNF-α, tumour necrosis factor-α; HMW, high molecular weight; HOMA2-IR, homeostasis model assessments of insulin resistance.

After adjusting for age, physical activity, fasting time, and BMI, plasma BCAA concentrations were significantly and inversely associated with total and HMW adiponectin levels and positively associated with leptin and C-peptide levels in both men (Table 3) and women (Table 4). HOMA2-IR was also positively associated with BCAA levels in participants fasting for 8 hours or more (Tables 3 and 4). In women, adipokine levels were higher than in men; however, the associations between BCAA levels and adipokines were similar for both sexes. Sensitivity analyses excluding participants with fasting times <8 h and those with HMW adiponectin and leptin levels below the level of detection limit were conducted. Moreover, stratified analyses of BMI for both sexes were performed. The results of the sensitivity and stratified analyses were nearly unchanged from the main results (data not shown in tables). The models for isoleucine, leucine, and valine and adipokines are shown in Supplementary Tables 1 (men) 2 (women). In both tables, the associations between the BCAA and adipokine levels were similar.

**Table 3:** Geometric means and 95% confidence intervals (CI) of adipokine concentrations and insulin-related markers, according to plasma branched-chain amino acid (BCAA) level quartile, in 435 men.

	Plasma BCAA quartile				P for trend^3^
	Q1 (n = 109)	Q2 (n = 109)	Q3 (n = 109)	Q4 (n = 108)	
BCAA (μmol/L)	≤360.0	396.1–430.9	430.9–475.9	≥ 476.1	
Total adiponectin (μg/mL)					
Crude	5.24 (4.81–5.69)	4.59 (4.22–4.99)	4.28 (3.94–4.66)	3.68 (3.38–4.00)	<0.0001
Adjusted 1^1^	5.24 (4.82–5.69)	4.66 (4.29–5.06)	4.22 (3.88–4.58)	3.67 (3.37–3.98)	<0.0001
Adjusted 2^2^	5.00 (4.60–5.44)	4.65 (4.29–5.04)	4.28 (3.94–4.64)	3.80 (3.50–4.13)	<0.0001
HMW adiponectin (μg/mL)					
Crude	1.62 (1.38–1.90)	1.39 (1.18–1.63)	1.17 (1.00–1.38)	0.86 (0.73–1.01)	<0.0001
Adjusted 1^1^	1.61 (1.38–1.89)	1.41 (1.21–1.66)	1.15 (0.98–1.35)	0.85 (0.73–1.00)	<0.0001
Adjusted 2^2^	1.51 (1.28–1.77)	1.41 (1.21–1.65)	1.17 (1.00–1.37)	0.91 (0.77–1.06)	<0.0001
Leptin (pg/mL)					
Crude	1124 (887–1424)	1946 (1536–2466)	2370 (1870–3003)	3451 (2720–4377)	<0.0001
Adjusted 1^1^	1150 (907–1460)	1896 (1495–2405)	2357 (1856–2994)	3505 (2760–4453)	<0.0001
Adjusted 2^2^	1681 (1359–2078)	1902 (1552–2331)	2130 (1736–2614)	2620 (2126–3228)	0.003
TNF-α (pg/mL)					
Crude	2.60 (2.41–2.81)	2.67 (2.48–2.88)	2.52 (2.33–2.71)	2.97 (2.75–3.20)	0.04
Adjusted 1^1^	2.59 (2.40–2.79)	2.69 (2.49–2.90)	2.50 (2.32–2.70)	2.96 (2.74–3.20)	0.04
Adjusted 2^2^	2.67 (2.47–2.89)	2.69 (2.49–2.90)	2.48 (2.30–2.68)	2.89 (2.68–3.13)	0.30
C-peptide (ng/mL)					
Crude	1.05 (0.97–1.12)	1.19 (1.11–1.28)	1.29 (1.20–1.38)	1.54 (1.44–1.66)	<0.0001
Adjusted 1^1^	1.05 (0.98–1.13)	1.18 (1.10–1.27)	1.30 (1.21–1.39)	1.55 (1.44–1.66)	<0.0001
Adjusted 2^2^	1.15 (1.08–1.23)	1.19 (1.11–1.26)	1.26 (1.18–1.35)	1.43 (1.34–1.53)	<0.0001
HOMA2-IR^3^					
Crude	0.81 (0.74–0.88)	0.87 (0.80–0.96)	0.99 (0.90–1.08)	1.20 (1.10–1.30)	<0.0001
Adjusted 1^3^	0.81 (0.74–0.88)	0.87 (0.79–0.95)	0.99 (0.91–1.08)	1.21 (1.11–1.31)	<0.0001
Adjusted 2^3^	0.89 (0.82–0.96)	0.88 (0.81–0.96)	0.96 (0.88–1.04)	1.11 (1.03–1.21)	<0.0001

Except for BCAA and *P* for trend, values are expressed as geometric means (95% CI).

^1^Adjusted for age (continuous), physical activity (quartile), and fasting time (<8 h, > 8 h).

^2^Further adjusted for body mass index (continuous).

^3^HOMA2-IR was calculated in participants with 8 hours or more fasting (n = 298). Number of participants in each BCAA category was 80 in Q1, 69 in Q2, 70 in Q3 and 79 in Q4. “Adjusted 1” model was adjusted for age (continuous) and physical activity (quartile). “Adjusted 2” model was further adjusted for body mass index (continuous).

^4^Median adipokine values for each category were used to test linear trends.

HMW, high molecular weight; HOMA2-IR, homeostasis model assessments of insulin resistance.

**Table 4:** Geometric means and 95% confidence intervals (CIs) of adipokine concentrations and insulin-related markers, according to plasma branched-chain amino acid (BCAA) level quartile, in 243 women.

BCAA (μmol/L)	Plasma BCAA quartile category				P for trend^4^
	Q1 (n = 61)	Q2 (n = 61)	Q3 (n = 61)	Q4 (n = 60)	
	≤316.7	316.8–345.8	345.8–388.4	≥388.5	
Total adiponectin (μg/mL)					
Crude	8.33 (7.49–9.26)	6.88 (6.19–7.65)	6.72 (6.05–7.47)	5.96 (5.36–6.63)	<0.0001
Adjusted 1^1^	8.35 (7.52–9.27)	6.98 (6.29–7.75)	6.75 (6.07–7.50)	5.86 (5.27–6.52)	<0.0001
Adjusted 2^2^	8.16 (7.33–9.08)	6.92 (6.23–7.68)	6.84 (6.15–7.61)	5.98 (5.36–6.66)	0.0002
HMW adiponectin (μg/mL)					
Crude	3.73 (3.18–4.39)	2.73 (2.33–3.21)	2.64 (2.25–3.11)	2.25 (1.91–2.64)	<0.0001
Adjusted 1^1^	3.74 (3.19–4.40)	2.78 (2.37–3.26)	2.67 (2.27–3.14)	2.22 (1.88–2.62)	<0.0001
Adjusted 2^2^	3.61 (3.06–4.26)	2.74 (2.33–3.22)	2.73 (2.31–3.21)	2.29 (1.94–2.70)	0.0005
Leptin (pg/mL)					
Crude	3355 (2725–4130)	5031 (4087–6193)	5931 (4818–7302)	7810 (6333–9631)	<0.0001
Adjusted 1^1^	3378 (2740–4164)	5026 (4078–6195)	5975 (4831–7389)	7919 (6392–9812)	<0.0001
Adjusted 2^2^	4270 (3583–5088)	5495 (4632–6520)	5201 (4368–6194)	6510 (5449–7778)	0.003
TNF-α (pg/mL)					
Crude	2.34 (2.16–2.54)	2.39 (2.20–2.59)	2.52 (2.32–2.73)	2.63 (2.42–2.85)	0.03
Adjusted 1^1^	2.35 (2.17–2.54)	2.41 (2.22–2.61)	2.52 (2.33–2.73)	2.55 (2.35–2.76)	0.12
Adjusted 2^2^	2.35 (2.16–2.55)	2.41 (2.22–2.61)	2.52 (2.32–2.73)	2.55 (2.34–2.77)	0.15
C-peptide (ng/mL)					
Crude	1.02 (0.94–1.10)	1.05 (0.97–1.13)	1.08 (1.00–1.16)	1.25 (1.15–1.35)	0.0002
Adjusted 1^1^	1.00 (0.93–1.08)	1.04 (0.97–1.13)	1.08 (1.00–1.17)	1.26 (1.17–1.36)	<0.0001
Adjusted 2^2^	1.05 (0.97–1.13)	1.06 (0.98–1.14)	1.05 (0.98–1.14)	1.22 (1.13–1.31)	0.01
HOMA2-IR^3^					
Crude	0.76 (0.70–0.83)	0.79 (0.73–0.84)	0.83 (0.76–0.90)	0.99 (0.90–1.08)	<0.0001
Adjusted 1^1^	0.76 (0.70–0.82)	0.79 (0.72–0.86)	0.84 (0.77–0.91)	0.99 (0.90–1.08)	<0.0001
Adjusted 2^2^	0.79 (0.72–0.86)	0.80 (0.74–0.87)	0.81 (0.74–0.88)	0.96 (0.87–1.05)	0.004

Except BCAA and *P* for trend, values are expressed as geometric means (95% CI).

^1^Adjusted for age (continuous), physical activity (quartile) and fasting time (<8 h, >8 h).

^2^Further adjusted for body mass index (continuous).

^3^HOMA2-IR was calculated in participants with 8 hours or more fasting (n = 208). Number of participants in each BCAA category was 55 in Q1, 53 in Q2, 53 in Q3 and 47 in Q4. “Adjusted 1” model was adjusted for age (continuous) and physical activity (quartile). “Adjusted 2” model was further adjusted for body mass index (continuous).

^4^Median adipokine values for each category were used to test linear trends.

HMW, high molecular weight, HOMA2-IR, homeostasis model assessments of insulin resistance.

## Discussion

In this study, high plasma concentrations of BCAAs were associated with low total and HMW adiponectin levels and with high leptin concentrations in non-diabetic Japanese men and women, after adjusting for age, fasting time, physical activity, and BMI. We also confirmed a positive association of BCAA with C-peptide concentrations and HOMA2-IR. The results of this study suggest a new insight into the interrelationship among plasma BCAAs, adipokines, and insulin resistance; previous reports have focused mainly on the adipokine—insulin resistance and BCAA—insulin resistance relationships^3–8,12^.

We found that BCAA concentrations are inversely associated with total and HMW adiponectin levels and are positively associated with leptin levels in both men and women. A previous study among patients with diabetes reported an association between BCAA levels and adipokines; however, it only reported the inverse association between BCAA and adiponectin concentrations, but did not report a significant correlation between BCAA and leptin levels^14^. The inconsistent results for the leptin association might be explained by differences in the participant characteristics between this study and that of previous studies. The previous study was limited by a small sample size (50 subjects), the absence of a sex-specific analysis, and no adjustment for potential confounding factors (e.g., BMI). Moreover, since “leptin resistance” is known to occur in obese individuals^26^, patients with diabetes might demonstrate different associations between leptin and BCAA levels than do individuals without diabetes.

Several studies have reported that BCAA concentrations are associated with poor metabolic health, including insulin resistance^10–13^. Several factors including intrinsic genetic variation, obesity, and adipose tissue distribution were reported to be associated with regulation of BCAA levels^27–30^. Genetic predisposition to BCAA levels was shown from genome-wide association studies, in which genetic correlation of BCAA levels with insulin resistance, and the association between predisposition to higher BCAA levels and increased risk of type 2 diabetes were reported^27^. Moreover, impaired BCAA metabolism may partially explain the association between adiposity and insulin resistance, because adipose tissue affects catabolism of BCAA^28,30^. Several underlying mechanisms between plasma BCAA levels and insulin resistance, including the mammalian target of rapamycin complex 1 (mTORC1) pathway, have been suggested; that is, increased BCAA concentrations caused by excess nutrient intake activates mTORC1 and causes insulin resistance through the insulin receptor substrate^28,29^. However, other common factors directly linking BCAA levels and insulin resistance remain to be clarified^28,29^. Our results add a new, hypothetical pathway between BCAA levels and insulin resistance, via adipokines. Specifically, BCAAs might alter some adipocytes, thereby regulating the adipokines that cause insulin resistance. Otherwise, the association between BCAA levels and adipokines might be merely stemming from residual confounding, in the absence of a direct connection between them. Moreover, reverse causality might explain the association; specifically, a change in BCAA levels might be the result of adipokine changes. Indeed, Liu *et al*. reported that adiponectins corrected the altered BCAA metabolism induced by high fat diets in mice, and Lian *et al*. also found that decreases in adiponectin signalling was associated with a reduction in enzyme activity involved in BCAA catabolism^31,32^. Although our results show a significant association between BCAA levels and adipokines, regardless of adiposity, whether adipokines have an intermediary role in connecting BCAA levels with insulin resistance will require further investigation.

Some of the limitations of this study should be described. First, this was a cross-sectional study, meaning that temporal relationships could not be established. Second, there may be unknown confounding factors. In this study, we selected potential confounding factors according to the studies already reported; however, the possibility of residual confounding remains. Third, the participants also comprised the control group of individuals in a cancer screening program. Men over 50-years-old and women over 40-years-old were included and may represent individuals who are more health conscious than the general Japanese population. In the 2004 National Health and Nutrition Survey in Japan, the proportion of people with BMIs > 25 included approximately 25–30% of men and 20–30% of women in these age ranges^33^. Compared to this, the average BMI was lower for our participants than for the general Japanese population. To improve the generalisation of the present results, younger individuals and those with higher BMIs should be assessed in future studies.

In conclusion, the positive association between plasma BCAA and leptin levels, and the inverse association between BCAA and adiponectin, including HMW adiponectin, levels were identified, after adjusting for potential confounding factors. Additional studies are required to confirm these results and reveal the mechanisms underlying these associations.

## Electronic supplementary material

Supplemental tables
