# Supplementary material for: Association between plasma concentrations of branched-chain amino acids and adipokines in Japanese adults without diabetes
Source: Sci Rep. 2018 Jan 18;8:1043. doi: 10.1038/s41598-018-19388-w (PMC5773488; doi:10.1038/s41598-018-19388-w)
Supplement: Supplementary file 1 — Supplemental tables [file 41598_2018_19388_MOESM1_ESM.doc]

Title:　Association between plasma concentrations of branched-chain amino acids and adipokines in Japanese adults without diabetes

**Authors**: Ryoko Katagiri, Atsushi Goto, Sanjeev Budhathoki, Taiki Yamaji, Hiroshi Yamamoto, Yumiko Kato, Motoki Iwasaki, Shoichiro Tsugane

<Supplemental tables>

Supplimentary Table S1. Geometric means and 95% confidence intervals (CIs) of concentrations of adipokines and insulin-related markers, according to plasma amino acid level quartile, in 435 men

|  | Plasma amino acid level quartile | | | |  |
| --- | --- | --- | --- | --- | --- |
|  | Q1  (n = 109) | Q2  (n = 109) | Q3  (n = 109) | Q4  (n = 108) | P for trend^4^ |
| Isoleucine (μmol/L) | ≤58.1 | 58.1–65.5 | 65.5–73.3 | ≥73.3 |  |
| Total adiponectin (μg/mL) |  |  |  |  |  |
| Crude | 5.21 (4.79–5.66) | 4.60 (4.24–5.00) | 4.50 (4.14–4.89) | 3.50 (3.22–3.81) | ＜0.0001 |
| Adjusted 1^1^ | 5.16 (4.76–5.60) | 4.67 (4.31–5.07) | 4.46 (4.11–4.84) | 3.52 (3.24–3.82) | ＜0.0001 |
| Adjusted 2^2^ | 4.97 (4.58–5.40) | 4.60 (4.24–4.98) | 4.54 (4.19–4.92) | 3.65 (3.36–3.96) | ＜0.0001 |
|  |  |  |  |  |  |
| HMW adiponectin (μg/mL) |  |  |  |  |  |
| Crude | 1.62 (1.38–1.89) | 1.36 (1.16–1.59) | 1.30 (1.11–1.52) | 0.79 (0.67–0.93) | ＜0.0001 |
| Adjusted 1^1^ | 1.58 (1.36–1.85) | 1.40 (1.20–1.64) | 1.27 (1.09–1.49) | 0.80 (0.68–0.93) | < 0.0001 |
| Adjusted 2^2^ | 1.49 (1.28–1.75) | 1.37 (1.17–1.60) | 1.31 (1.12–1.53) | 0.84 (0.72–0.99) | < 0.0001 |
|  |  |  |  |  |  |
| Leptin (pg/mL) |  |  |  |  |  |
| Crude | 1192 (939–1512) | 1795 (1414–2278) | 2571 (2026–3263) | 3250 (2558–4129) | ＜0.0001 |
| Adjusted 1^1^ | 1212 (955–1539) | 1757 (1384–2232) | 2569 (2024–3261) | 3266 (2572–4148) | ＜0.0001 |
| Adjusted 2^2^ | 1636 (1328–2016) | 1998 (1628–2452) | 2213 (1804–2716) | 2464 (2001–3034) | 0.007 |
|  |  |  |  |  |  |
| TNF-α (pg/mL) |  |  |  |  |  |
| Crude | 2.63 (2.44–2.84) | 2.58 (2.39–2.88) | 2.69 (2.49–2.90) | 2.83 (2.63–3.06) | 0.12 |
| Adjusted 1^1^ | 2.59 (2.40–2.80) | 2.61 (2.42–2.82) | 2.67 (2.48–2.89) | 2.85 (2.64–3.07) | 0.08 |
| Adjusted 2^2^ | 2.66 (2.46–2.87) | 2.64 (2.45–2.85) | 2.64 (2.45–2.85) | 2.78 (2.58–3.01) | 0.42 |
|  |  |  |  |  |  |
| C-peptide (ng/mL) |  |  |  |  |  |
| Crude | 1.07 (0.99–1.15) | 1.17 (1.09–1.25) | 1.30 (1.21–1.40) | 1.53 (1.42–1.64) | ＜0.0001 |
| Adjusted 1^1^ | 1.07 (1.00–1.15) | 1.16 (1.08–1.24) | 1.31 (1.22–1.41) | 1.53 (1.42–1.64) | ＜0.0001 |
| Adjusted 2^2^ | 1.16 (1.08–1.23) | 1.20 (1.12–1.28) | 1.26 (1.18–1.34) | 1.42 (1.33–1.51) | ＜0.0001 |
|  |  |  |  |  |  |
| HOMA2-IR^3^ |  |  |  |  |  |
| Crude | 0.81 (0.74–0.88) | 0.88 (0.81–0.96) | 0.97 (0.89–1.06) | 1.21 (1.11–1.31) | ＜0.0001 |
| Adjusted 1^3^ | 0.81 (0.75–0.89) | 0.86 (0.80–0.96) | 0.98 (0.89–1.07) | 1.21 (1.11–1.32) | ＜0.0001 |
| Adjusted 2^3^ | 0.88 (0.81–0.96) | 0.90 (0.83–0.98) | 0.95 (0.87–1.03) | 1.12 (1.03–1.21) | ＜0.0001 |
| Leucine (μmol/L) | ≤118.0 | 118.0–129.5 | 129.5–142.7 | 142.8< |  |
| Total adiponectin (μg/mL) |  |  |  |  |  |
| Crude | 5.17 (4.75–5.62) | 4.86 (4.47–5.29) | 4.01 (3.69–4.36) | 3.75 (3.45–4.08) | ＜0.0001 |
| Adjusted 1^1^ | 5.14 (4.73–5.57) | 4.94 (4.56–5.36) | 4.05 (3.73–4.39) | 3.68 (3.39–3.99) | ＜0.0001 |
| Adjusted 2^2^ | 4.91 (4.52–5.34) | 4.90 (4.52–5.31) | 4.11 (3.79–4.45) | 3.83 (3.52–4.16) | ＜0.0001 |
|  |  |  |  |  |  |
| HMW adiponectin (μg/mL) |  |  |  |  |  |
| Crude | 1.61 (1.38–1.89) | 1.48 (1.26–1.74) | 1.05 (0.90–1.24) | 0.90 (0.76–1.05) | ＜0.0001 |
| Adjusted 1^1^ | 1.59 (1.36–1.86) | 1.53 (1.31–1.79) | 1.08 (0.92–1.26) | 0.86 (0.74–1.01) | < 0.0001 |
| Adjusted 2^2^ | 1.49 (1.27–1.76) | 1.51 (1.29–1.76) | 1.10 (0.94–1.29) | 0.92 (0.78–1.08) | < 0.0001 |
|  |  |  |  |  |  |
| Leptin (pg/mL) |  |  |  |  |  |
| Crude | 1401 (1100–1784) | 1732 (1360–2206) | 2260 (1775–2877) | 3259 (2557–4154) | ＜0.0001 |
| Adjusted 1^1^ | 1406 (1105–1789) | 1702 (1338–2167) | 2227 (1750–2834) | 3354 (2630–4276) | ＜0.0001 |
| Adjusted 2^2^ | 2063 (1668–2551) | 1844 (1503–2263) | 1976 (1609–2425) | 2372 (1918–2933) | 0.33 |
|  |  |  |  |  |  |
| TNF-α (pg/mL) |  |  |  |  |  |
| Crude | 2.65 (2.45–2.86) | 2.57 (2.39–2.78) | 2.60 (2.41–2.81) | 2.92 (2.71–3.16) | 0.07 |
| Adjusted 1^1^ | 2.62 (2.43–2.83) | 2.59 (2.40–2.79) | 2.62 (2.43–2.83) | 2.91 (2.69–3.14) | 0.06 |
| Adjusted 2^2^ | 2.70 (2.49–2.92) | 2.61 (2.42–2.81) | 2.60 (2.41–2.80) | 2.83 (2.61–3.06) | 0.43 |
|  |  |  |  |  |  |
| C-peptide (ng/mL) |  |  |  |  |  |
| Crude | 1.13 (1.05–1.21) | 1.13 (1.05–1.22) | 1.32 (1.23–1.42) | 1.47 (1.37–1.58) | ＜0.0001 |
| Adjusted 1^1^ | 1.13 (1.05–1.21) | 1.12 (1.04–1.21) | 1.31 (1.22–1.41) | 1.49 (1.38–1.60) | ＜0.0001 |
| Adjusted 2^2^ | 1.25 (1.17–1.34) | 1.15 (1.08–1.22) | 1.27 (1.19–1.36) | 1.36 (1.27–1.45) | 0.03 |
|  |  |  |  |  |  |
| HOMA2-IR^3^ |  |  |  |  |  |
| Crude | 0.83 (0.76–0.91) | 0.86 (0.79–0.94) | 1.03 (0.94–1.13) | 1.13 (1.04–1.23) | ＜0.0001 |
| Adjusted 1^3^ | 0.83 (0.76–0.91) | 0.85 (0.78–0.93) | 1.02 (0.93–1.12) | 1.15 (1.05–1.25) | ＜0.0001 |
| Adjusted 2^3^ | 0.92 (0.85–1.00) | 0.89 (0.82–0.97) | 0.98 (0.90–1.06) | 1.04 (0.96–1.13) | 0.02 |
| Valine (μmol/L) | ≤216.5 | 216.5–237.1 | 237.2–262.9 | 263.0< |  |
| Total adiponectin (μg/mL) |  |  |  |  |  |
| Crude | 5.00 (4.60–5.45) | 4.79 (4.40–5.21) | 4.19 (3.85–4.56) | 3.77 (3.46–4.10) | ＜0.0001 |
| Adjusted 1^1^ | 5.06 (4.66–5.50) | 4.79 (4.41–5.20) | 4.17 (3.83–4.53) | 3.75 (3.45–4.07) | ＜0.0001 |
| Adjusted 2^2^ | 4.84 (4.45–5.26) | 4.77 (4.40–5.17) | 4.24 (3.90–4.60) | 3.87 (3.56–4.21) | ＜0.0001 |
|  |  |  |  |  |  |
| HMW adiponectin (μg/mL) |  |  |  |  |  |
| Crude | 1.52 (1.29–1.79) | 1.46 (1.24–1.71) | 1.14 (0.97–1.34) | 0.90 (0.76–1.05) | ＜0.0001 |
| Adjusted 1^1^ | 1.55 (1.32–1.82) | 1.45 (1.23–1.70) | 1.13 (0.97–1.33) | 0.89 (0.76–1.04) | ＜0.0001 |
| Adjusted 2^2^ | 1.45 (1.23–1.70) | 1.44 (1.23–1.68) | 1.16 (0.99–1.36) | 0.94 (0.80–1.10) | ＜0.0001 |
|  |  |  |  |  |  |
| Leptin (pg/mL) |  |  |  |  |  |
| Crude | 1186 (933–1506) | 2066 (1626–2624) | 2270 (1787–2885) | 3214 (2527–4088) | ＜0.0001 |
| Adjusted 1^1^ | 1190 (937–1512) | 2009 (1580–2553) | 2285 (1796–2908) | 3272 (2572–4163) | ＜0.0001 |
| Adjusted 2^2^ | 1660 (1346–2047) | 2076 (1693–2545) | 2015 (1641–2475) | 2569 (2087–3162) | 0.007 |
|  |  |  |  |  |  |
| TNF-α (pg/mL) |  |  |  |  |  |
| Crude | 2.66 (2.46–2.87) | 2.61 (2.42–2.81) | 2.60 (2.41–2.81) | 2.88 (2.67–3.11) | 0.14 |
| Adjusted 1^1^ | 2.66 (2.46–2.87) | 2.60 (2.41–2.81) | 2.60 (2.40–2.80) | 2.87 (2.66–3.10) | 0.16 |
| Adjusted 2^2^ | 2.73 (2.53–2.96) | 2.61 (2.42–2.82) | 2.57 (2.38–2.77) | 2.81 (2.60–3.04) | 0.61 |
|  |  |  |  |  |  |
| C-peptide (ng/mL) |  |  |  |  |  |
| Crude | 1.04 (0.97–1.12) | 1.21 (1.12–1.29) | 1.30 (1.21–1.39) | 1.52 (1.42–1.64) | ＜0.0001 |
| Adjusted 1^1^ | 1.03 (0.96–1.11) | 1.21 (1.13–1.30) | 1.30 (1.21–1.40) | 1.52 (1.41–1.63) | ＜0.0001 |
| Adjusted 2^2^ | 1.12 (1.05–1.20) | 1.22 (1.15–1.30) | 1.26 (1.18–1.34) | 1.43 (1.34–1.52) | ＜0.0001 |
|  |  |  |  |  |  |
| HOMA2-IR^3^ |  |  |  |  |  |
| Crude | 0.80 (0.73–0.87) | 0.92 (0.84–1.01) | 0.97 (0.88–1.06) | 1.18 (1.09–1.29) | ＜0.0001 |
| Adjusted 1^3^ | 0.79 (0.73–0.86) | 0.92 (0.84–1.00) | 0.96 (0.88–1.05) | 1.19 (1.10–1.30) | ＜0.0001 |
| Adjusted 2^3^ | 0.87 (0.80–0.94) | 0.93 (0.86–1.01) | 0.93 (0.86–1.01) | 1.11 (1.03–1.20) | ＜0.0001 |

Except for BCAA and *P* for trend, values are expressed as geometric means (95% CI).

1. Adjusted for age (continuous), physical activity (quartile), and fasting time (<8h, >8h).

2. Further adjusted for body mass index (continuous).

3. HOMA2-IR was calculated in participants with 8 hours or more fasting (n=298). Number of participants in each BCAA category was 80 in Q1, 69 in Q2, 70 in Q3 and 79 in Q4. “Adjusted 1” model was adjusted for age (continuous) and physical activity (quartile). “Adjusted 2” model was further adjusted for body mass index (continuous).

4. Median adipokine values for each category were used to test linear trends.

HMW, high molecular weight

Supplementary Table S2. Geometric means and 95% confidence intervals (CI) of concentrations of adipokines and insulin-related markers, according to plasma branched-chain amino acid (BCAA) level quartiles, in 243 women

|  | BCAA level quartile | | | |  |
| --- | --- | --- | --- | --- | --- |
|  | Q1  (n = 61) | Q2  (n = 61) | Q3  (n = 61) | Q4  (n = 60) | P for trend^4^ |
| Isoleucine (μmol/L) | ≤44.7 | 44.8–50.1 | 50.2–56.8 | ≥56.9 |  |
| Total adiponectin (μg/mL) |  |  |  |  |  |
| Crude | 8.34 (7.50–9.26) | 7.09 (6.38–7.88) | 6.02 (5.41–6.69) | 6.47 (5.81–7.19) | 0.0003 |
| Adjusted 1^1^ | 8.41 (7.56–9.34) | 7.09 (6.39–7.88) | 6.07 (5.46–6.74) | 6.37 (5.71–7.11) | 0.0001 |
| Adjusted 2^2^ | 8.21 (7.37–9.15) | 7.00 (6.30–7.77) | 6.14 (5.52–6.82) | 6.54 (5.84–7.32) | 0.003 |
|  |  |  |  |  |  |
| HMW adiponectin (μg/mL) |  |  |  |  |  |
| Crude | 3.68 (3.13–4.33) | 2.76 (2.34–3.24) | 2.41 (2.05–2.84) | 2.48 (2.10–2.92) | 0.0007 |
| Adjusted 1^1^ | 3.73 (3.17–4.40) | 2.76 (2.35–3.24) | 2.44 (2.07–2.88) | 2.45 (2.07–2.90) | 0.0005 |
| Adjusted 2^2^ | 3.59 (3.04–4.25) | 2.70 (2.30–3.18) | 2.49 (2.11–2.93) | 2.56 (2.15–3.04) | 0.01 |
|  |  |  |  |  |  |
| Leptin (pg/mL) |  |  |  |  |  |
| Crude | 3734 (3019–4619) | 4655 (3763–5757) | 6520 (5271–8064) | 6884 (5556–8530) | ＜0.0001 |
| Adjusted 1^1^ | 3751 (3019–4660) | 4685 (3783–5802) | 6471 (5212–8034) | 7067 (5647–8845) | ＜0.0001 |
| Adjusted 2^2^ | 4742 (3959–5681) | 5334 (4479–6352) | 5731 (4804–6838) | 5462 (4529–6588) | 0.27 |
|  |  |  |  |  |  |
| TNF-α (pg/mL) |  |  |  |  |  |
| Crude | 2.34 (2.15–2.54) | 2.44 (2.25–2.65) | 2.53 (2.33–2.75) | 2.56 (2.36–2.78) | 0.10 |
| Adjusted 1^1^ | 2.34 (2.16–2.54) | 2.44 (2.26–2.64) | 2.55 (2.36–2.76) | 2.49 (2.29–2.70) | 0.23 |
| Adjusted 2^2^ | 2.34 (2.16–2.55) | 2.44 (2.26–2.65) | 2.55 (2.35–2.76) | 2.48 (2.28–2.70) | 0.31 |
|  |  |  |  |  |  |
| C-peptide (ng/mL) |  |  |  |  |  |
| Crude | 0.99 (0.92–1.07) | 1.07 (0.99–1.16) | 1.16 (1.07–1.26) | 1.15 (1.07–1.25) | 0.004 |
| Adjusted 1^1^ | 0.97 (0.89–1.05) | 1.08 (1.00–1.16) | 1.15 (1.07–1.25) | 1.19 (1.10–1.29) | 0.0002 |
| Adjusted 2^2^ | 1.01 (0.93–1.09) | 1.10 (1.02–1.19) | 1.14 (1.05–1.23) | 1.14 (1.05–1.23) | 0.04 |
|  |  |  |  |  |  |
| HOMA2-IR^3^ |  |  |  |  |  |
| Crude | 0.75 (0.69–0.82) | 0.80 (0.74–0.87) | 0.89 (0.82–0.97) | 0.92 (0.83–1.01) | 0.0005 |
| Adjusted 1^3^ | 0.75 (0.69–0.81) | 0.81 (0.74–0.88) | 0.89 (0.82–0.97) | 0.93 (0.84–1.02) | 0.0002 |
| Adjusted 2^3^ | 0.77 (0.71–0.84) | 0.83 (0.76–0.90) | 0.87 (0.74–0.88) | 0.96 (0.87–1.05) | 0.03 |
| Leucine (μmol/L) | ≤91.7 | 91.8–102.8 | 102.9–114.2 | ≥114.5 |  |
| Total adiponectin (μg/mL) |  |  |  |  |  |
| Crude | 8.43 (7.59–9.37) | 6.88 (6.19–7.64) | 6.68 (6.01–7.42) | 5.92 (5.33–6.59) | ＜0.0001 |
| Adjusted 1^1^ | 8.36 (7.53–9.28) | 7.04 (6.34–7.82) | 6.67 (6.01–7.41) | 5.85 (5.26–6.52) | ＜0.0001 |
| Adjusted 2^2^ | 8.17 (7.33–9.10) | 7.03 (6.33–7.80) | 6.69 (6.03–7.43) | 5.99 (5.36–6.69) | 0.0002 |
|  |  |  |  |  |  |
| HMW adiponectin (μg/mL) |  |  |  |  |  |
| Crude | 3.78 (3.22–4.44) | 2.74 (2.34–3.22) | 2.72 (2.31–3.19) | 2.15 (1.83–2.52) | ＜0.0001 |
| Adjusted 1^1^ | 3.75 (3.20–4.40) | 2.82 (2.40–3.31) | 2.71 (2.31–3.19) | 2.14 (1.82–2.52) | < 0.0001 |
| Adjusted 2^2^ | 3.63 (3.07–4.28) | 2.81 (2.39–3.30) | 2.73 (2.32–3.20) | 2.21 (1.87–2.62) | 0.0001 |
|  |  |  |  |  |  |
| Leptin (pg/mL) |  |  |  |  |  |
| Crude | 3497 (2834–4315) | 5313 (4306–6555) | 5435 (4405–6706) | 7740 (6262–9566) | ＜0.0001 |
| Adjusted 1^1^ | 3478 (2816–4295) | 5380 (4351–6652) | 5421 (4387–6700) | 7945 (6396–9868) | ＜0.0001 |
| Adjusted 2^2^ | 4515 (3775–5400) | 5519 (4640–6564) | 5290 (4364–6171) | 6149 (5120–7385) | 0.04 |
|  |  |  |  |  |  |
| TNF-α (pg/mL) |  |  |  |  |  |
| Crude | 2.43 (2.24–2.63) | 2.30 (2.12–2.49) | 2.55 (2.35–2.77) | 2.60 (2.40–2.82) | 0.09 |
| Adjusted 1^1^ | 2.40 (2.22–2.60) | 2.35 (2.17–2.54) | 2.55 (2.36–2.76) | 2.53 (2.33–2.74) | 0.21 |
| Adjusted 2^2^ | 2.41 (2.22–2.62) | 2.35 (2.17–2.54) | 2.55 (2.35–2.76) | 2.52 (2.31–2.74) | 0.28 |
|  |  |  |  |  |  |
| C-peptide (ng/mL) |  |  |  |  |  |
| Crude | 1.03 (0.95–1.12) | 1.06 (0.98–1.15) | 1.10 (1.02–1.19) | 1.19 (1.10–1.29) | 0.009 |
| Adjusted 1^1^ | 1.01 (0.94–1.10) | 1.07 (0.99–1.16) | 1.09 (1.01–1.18) | 1.21 (1.11–1.31) | 0.003 |
| Adjusted 2^2^ | 1.07 (0.99–1.15) | 1.08 (1.00–1.16) | 1.08 (1.00–1.17) | 1.15 (1.06–1.25) | 0.20 |
|  |  |  |  |  |  |
| HOMA2-IR^3^ |  |  |  |  |  |
| Crude | 0.78 (0.71–0.84) | 0.81 (0.74–0.88) | 0.84 (0.77–0.92) | 0.92 (0.84–1.00) | 0.006 |
| Adjusted 1^3^ | 0.77 (0.71–0.84) | 0.82 (0.75–0.89) | 0.84 (0.77–0.91) | 0.93 (0.85–1.02) | 0.003 |
| Adjusted 2^3^ | 0.81 (0.74–0.88) | 0.82 (0.75–0.89) | 0.83 (0.76–0.90) | 0.88 (0.80–0.97) | 0.17 |
| Valine (μmol/L) | ≤176.1 | 176.3–193.4 | 193.6–220.0 | ≥220.3 |  |
| Total adiponectin (μg/mL) |  |  |  |  |  |
| Crude | 8.36 (7.53–9.28) | 6.72 (6.06–7.46) | 7.11 (6.40–7.89) | 5.75 (5.17–6.39) | ＜0.0001 |
| Adjusted 1^1^ | 8.35 (7.53–9.26) | 6.75 (6.09–7.48) | 7.24 (6.52–8.04) | 5.65 (5.09–6.28) | ＜0.0001 |
| Adjusted 2^2^ | 8.16 (7.36–9.05) | 6.67 (6.02–7.39) | 7.38 (6.64–8.19) | 5.75 (5.18–6.39) | ＜0.0001 |
|  |  |  |  |  |  |
| HMW adiponectin (μg/mL) |  |  |  |  |  |
| Crude | 3.83 (3.26–4.49) | 2.58 (2.20–3.03) | 2.88 (2.46–3.38) | 2.12 (1.81–2.49) | ＜0.0001 |
| Adjusted 1^1^ | 3.83 (3.27–4.48) | 2.59 (2.21–3.03) | 2.96 (2.52–3.48) | 2.10 (1.79–2.47) | < 0.0001 |
| Adjusted 2^2^ | 3.69 (3.15–4.33) | 2.55 (2.17–2.98) | 3.05 (2.59–3.58) | 2.16 (1.84–2.54) | < 0.0001 |
|  |  |  |  |  |  |
| Leptin (pg/mL) |  |  |  |  |  |
| Crude | 3483 (2825–4295) | 4754 (3855–5862) | 6448 (5229–7952) | 7313 (5920–9034) | ＜0.0001 |
| Adjusted 1^1^ | 3536 (2864–4367) | 4772 (3865–5892) | 6435 (5189–7980) | 7398 (5965–9175) | ＜0.0001 |
| Adjusted 2^2^ | 4286 (3602–5010) | 5265 (4437–6248) | 5523 (4632–6585) | 6383 (5354–7610) | 0.003 |
|  |  |  |  |  |  |
| TNF-α (pg/mL) |  |  |  |  |  |
| Crude | 2.32 (2.14–2.52) | 2.50 (2.31–2.71) | 2.45 (2.26–2.66) | 2.60 (2.39–2.82) | 0.09 |
| Adjusted 1^1^ | 2.33 (2.15–2.52) | 2.51 (2.32–2.72) | 2.46 (2.27–2.67) | 2.52 (2.32–2.73) | 0.26 |
| Adjusted 2^2^ | 2.34 (2.15–2.53) | 2.52 (2.33–2.73) | 2.45 (2.26–2.66) | 2.51 (2.31–2.73) | 0.32 |
|  |  |  |  |  |  |
| C-peptide (ng/mL) |  |  |  |  |  |
| Crude | 1.00 (0.92–1.08) | 1.02 (0.95–1.11) | 1.12 (1.03–1.21) | 1.25 (1.16–1.35) | ＜0.0001 |
| Adjusted 1^1^ | 0.99 (0.92–1.07) | 1.03 (0.96–1.11) | 1.11 (1.03–1.20) | 1.25 (1.16–1.36) | ＜0.0001 |
| Adjusted 2^2^ | 1.03 (0.95–1.11) | 1.05 (0.98–1.13) | 1.08 (1.00–1.17) | 1.22 (1.13–1.32) | 0.0009 |
|  |  |  |  |  |  |
| HOMA2-IR^3^ |  |  |  |  |  |
| Crude | 0.75 (0.69–0.82) | 0.78 (0.71–0.85) | 0.86 (0.79–0.93) | 0.96 (0.88–1.05) | ＜0.0001 |
| Adjusted 1^3^ | 0.75 (0.69–0.82) | 0.78 (0.72–0.85) | 0.86 (0.79–0.94) | 0.96 (0.88–1.05) | ＜0.0001 |
| Adjusted 2^3^ | 0.77 (0.71–0.84) | 0.80 (0.73–0.87) | 0.84 (0.77–0.91) | 0.94 (0.86–1.02) | 0.001 |

Except for BCAA and *P* for trend, values are expressed as geometric means (95% CI).

1. Adjusted for age (continuous), physical activity (quartile), and fasting time (<8h, >8h).

2. Further adjusted for body mass index (continuous).

3. HOMA2-IR was calculated in participants with 8 hours or more fasting (n=208). Number of participants in each BCAA category was 55 in Q1, 53 in Q2, 53 in Q3 and 47 in Q4. “Adjusted 1” model was adjusted for age (continuous) and physical activity (quartile). “Adjusted 2” model was further adjusted for body mass index (continuous).

4. Median adipokine values for each category were used to test linear trends.

HMW, high molecular weight
